# Clinical course, treatment and outcome of severe pneumocystis pneumonia in patients with autoimmune or inflammatory diseases: a prospective study

**DOI:** 10.3389/fcimb.2026.1715992

**Published:** 2026-05-27

**Authors:** Yan Shi, Na Wang, Xi Rui, Wei Liu, Hui You, Jin-min Peng

**Affiliations:** 1 Department of Intensive Care Unit, Peking Union Medical College Hospital, Peking Union Medical College and Chinese Academy of Medical Sciences, Beijing, China; 2 Department of Radiology, Peking Union Medical College Hospital, Peking Union Medical College and Chinese Academy of Medical Sciences, Beijing, China; 3 Department of Medical Intensive Care Unit, Peking Union Medical College Hospital, Peking Union Medical College and Chinese Academy of Medical Sciences, Beijing, China

**Keywords:** Acute respiratory failure, autoimmune or inflammatory disease, fibrosis scores, pneumocystis pneumonia, prognosis

## Abstract

**Background:**

The morbidity and mortality of *Pneumocystis* pneumonia (PCP) are rising among patients with autoimmune or inflammatory diseases (AID). Prospective studies focusing on severe PCP in this population remain scarce. A better understanding of the disease course and prognostic factors is urgently needed.

**Methods:**

A 5-year prospective single-center study in the ICU enrolled AID patients with severe PCP. Clinical, laboratory, radiological, therapeutic, and outcome data were collected prospectively. Multivariable regression was used to identify risk factors for initial treatment failure and 28-day ICU mortality.

**Results:**

Among 107 enrolled patients, the mean PaO_2_/FiO_2_ ratio was 167 mmHg, and 76.6% of patients required mechanical ventilation at ICU admission. Pulmonary coinfection was detected in 46 (43.0%) patients. Initial treatment failure occurred in 48.6% and was associated with underlying connective tissue disease-interstitial lung disease (OR 3.241, 95% CI 1.337-25.147, *p* = 0.004), coinfection (OR, 5.419; 95% CI, 1.565-38.759; *p* = 0.008), and severe hypoxemia on ICU admission (OR 6.873, 95% CI 1.746–31.048, *p* = 0.006). Follow-up pulmonary imaging revealed architectural distortions or worsening fibrosis in over 50% of patients. The 28-day ICU mortality was 57.0%; independently predicted by PaO_2_/FiO_2_ ratio < 100 mmHg at PCP onset (OR 8.119, 95% CI 1.543-32.871, *p* = 0.007), initial treatment failure (OR 10.591, 95% CI 1.926-45.768, *p* = 0.013), persistent CD_4_^+^ T-cell counts < 100 cells/μL (OR 4.137, 95% CI 1.219- 62.152, *p* = 0.021), fibrosis score > 260 during early follow-up (OR 5.354, 95% CI 1.513-19.756, *p* = 0.008), and new-onset shock during the ICU stay (OR 2.147, 95% CI 1.237-39.157, *p* = 0.018).

**Conclusions:**

PCP is life-threatening in AID patients. Beyond baseline hypoxemia, early follow-up variables, particularly initial treatment failure and progressive pulmonary fibrosis, are critical prognostic indicators, warranting further research to drive understanding of their causes.

## Introduction

*Pneumocystis* pneumonia (PCP) has increased markedly among non-HIV patients, with individuals suffering from autoimmune or inflammatory diseases (AID) accounting for over one-fifth of these cases (Fillatre et al., 2014; McMullan et al., 2024). However, few studies have specifically focused on the disease course and prognostic factors in this population. Most investigations have either incorporated AID patients as part of broader non-HIV PCP cohorts (Roux et al., 2014; Schmidt et al., 2018; Gaborit et al., 2019) or been limited to small-sample retrospective analyzes in this group (Aoki et al., 2009; Chen et al., 2015; Kageyama et al., 2019; Shi et al., 2025). Immune status and the nature of the underlying disease significantly influence the pathophysiology and mortality risk of PCP, as demonstrated by comparisons between HIV and non-HIV PCP patients (Ibrahim et al., 2023; McMullan et al., 2024). However, factors determining disease severity and mortality in the AID population remain poorly understood (Ibrahim et al., 2023). Recent evidence indicates that AID patients with PCP represent one of the most severely affected subgroups among non-HIV cases (Roblot et al., 2002; Kageyama et al., 2019; Lécuyer et al., 2024). It is plausible that immune dysregulation and multi-system involvement associated with AID may lead to a distinct clinical course in this cohort compared to other non-HIV PCP patients, although this hypothesis warrants further investigation.

Non-HIV PCP patients requiring intensive care unit (ICU) admission for acute respiratory failure carry a high mortality rates of up to 50% (Aoki et al., 2009; Schmidt et al., 2018; Gaborit et al., 2019). However, prospective studies focusing on severe PCP in AID patients are scarce. Moreover, existing prognostic analyzes have predominantly relied on baseline parameters (Aoki et al., 2009; Chen et al., 2015; Schmidt et al., 2018; Gaborit et al., 2019; Kageyama et al., 2019; Shi et al., 2025), whereas robust prognostic modeling should inherently incorporate both pretreatment parameters and dynamic variables emerging during antimicrobial therapy.

To address these gaps, we conducted a prospective cohort study of AID patients with severe PCP to comprehensively characterize the clinical course and identify predictors of outcomes, with particular focus on early follow-up variables, to help determine those patients most likely to benefit from intensive care.

## Methods

### Study design and participants

In this prospective cohort study, all adult AID patients hospitalized in the ICU of Peking Union Medical College Hospital (PUMCH) between July 2017 and July 2022 with suspected PCP were screened for enrollment. The initial microbiological workup for suspected pneumonia included staining, culture, antigen detection, serology, and nucleic acid amplification, as appropriate (**Supplementary Table 1**). The selection of subsequent pathogen tests and testing intervals was determined at the discretion of the attending clinicians. Patients meeting diagnostic criteria for severe PCP were included. Exclusion criteria included pregnancy, an established PCP diagnosis with more than 3 days of anti-PCP treatment prior to ICU admission, and an ICU length of stay (LOS) of less than 7 days (indicating insufficient data for treatment response evaluation). Additionally, during the COVID-19 pandemic, patients with confirmed SARS-CoV-2 infection were centrally managed at designated hospitals in accordance with institutional infection control policies (before December 2022). Therefore, patients with concomitant COVID-19 were not enrolled in this study.

This study was approved by the institutional review board of PUMCH and registered in Clinicaltrials.gov (NCT 03201497). The written informed consent was obtained from all participants or their relatives.

### Data collection and empirical anti-infective regimens

Demographic, comorbidities, and AID-specific treatments prior to ICU admission were prospectively collected. On ICU admission, disease severity was assessed by the Sequential Organ Failure Assessment (SOFA) and Acute Physiology and Chronic Health Evaluation (APACHE) II score, the PaO_2_/FiO_2_ (P/F) ratio, presence of septic shock, and need for mechanical ventilation (MV). Clinical, laboratory, and imaging data were obtained both at admission and after one week of anti-PCP treatment. Details of anti-PCP medications, corticosteroids adjunctive treatment (CAT), life-support interventions, complications, ICU LOS, and 28-day ICU mortality were recorded.

Regarding empirical anti-infective regimens at our institution, trimethoprim/sulfamethoxazole (TMP-SMX) is routinely used as the first-line treatment for PCP. Given the frequent coexistence of PCP and cytomegalovirus (CMV) infection, combined antiviral treatment constitutes a common empirical practice. In addition, pulmonary imaging abnormalities, including asymmetric ground-glass opacities, pulmonary masses, or consolidation, often prompt the initiation of empirical anti-aspergillus treatment. For community-acquired bacterial pneumonia, the standard regimen consists of ceftriaxone with or without moxifloxacin. Piperacillin/tazobactam or cefoperazone/sulbactam are the most frequently prescribed agents for hospital-acquired bacterial pneumonia.

### Radiological evaluation

High-resolution CT (HRCT) scans were performed at the time of PCP diagnosis and 7–10 days after initiation of anti-PCP treatment. All images were retrospectively and independently reviewed by two senior radiologists (WL and HY), who were blinded to clinical data. Fibrotic changes were quantified using a previously established scoring system, graded on a 1–6 scale as follows: 1, normal attenuation; 2, ground-glass attenuation; 3, consolidation; 4, ground-glass attenuation with traction bronchiolectasis or bronchiectasis; 5, consolidation with traction bronchiolectasis or bronchiectasis and 6, honeycombing. Each patterns was evaluated across three zones (upper, middle, lower) of each lung. The extent of each abnormality was determined by visually estimating the percentage (to the nearest 5%) of the affected lung parenchyma in each zone. The abnormality score per zone was calculated by multiplying the percentage area by the point value (1-6). The overall HRCT score for each patient was obtained by adding the six averaged scores (Fujimoto et al., 2012).

### Definitions

PCP diagnosis required typical clinical features, radiological findings and microbiological confirmation by methenamine silver stain or polymerase chain reaction (PCR) from respiratory specimens. Severe PCP was defined as the need for high-flow nasal cannula oxygen (HFNC) with FiO_2_ >50%, non-invasive ventilation (NIV), or MV (Gaborit et al., 2019). Pulmonary coinfection was defined by detection of additional pathogens in respiratory samples or serology at PCP onset. Invasive pulmonary fungal infection was diagnosed according to the consensus definitions of the European Organization of the Research and Treatment of Cancer/Mycoses Study Group, including proven and probable diagnosis (De Pauw et al., 2008). CMV pneumonia was presumptively diagnosed based on classic imaging findings and a high viral load by quantitative nucleic acid testing (QNAT) in bronchoalveolar lavage fluid (BALF) (Kotton et al., 2018). CAT referred to the intravenous administration of methylprednisolone at 40 mg twice per day from days 1 to 5, 40 mg once per day from days 6 to 10, and 20 mg once per day until day 21 or tapering to the pre-PCP maintenance dose. Initial treatment failure was defined as the absence of clinical improvement and/or worsening respiratory function per arterial blood gas analysis after 7 days of adequate anti-PCP therapy (Limper et al., 2011).

### Statistical analysis

Continuous data were compared using the Student’s t test or the Mann-Whitney U test. Categorical data were analyzed using the chi-square test or Fisher’s exact test as appropriate. Survival outcomes were assessed with Kaplan–Meier curves, and differences between groups were compared using the log-rank test. Prognostic factors were identified through logistic regression analysis using a stepwise backward elimination approach. Covariates with a *p* value < 0.1 in univariate analysis were included in the multivariate model. Univariate predictors consisted of baseline characteristics (e.g., demographics, comorbidities, type and treatment of AID, illness severity, coinfection status, radiological findings, laboratory parameters, and treatment for PCP), as well as follow-up variables (including initial treatment response, repeated laboratory results, radiological changes, and complications). All statistical analyzes were performed using SPSS software (version 22.0; IBM Inc., Armonk, NY). A two-sided *p* values < 0.05 was considered statistically significant.

## Results

### Study population and clinical features at ICU admission

During the study period, of 213 screened patients with suspected PCP, 153 were mycologically confirmed. After excluding 8 cases of *Pneumocystis* colonization, 4 cases who didn’t meet the diagnostic criteria for severe PCP, and 34 cases with an ICU LOS < 7 days, 107 patients (mean age 52 ± 17 years; 63.6% female) were included in the final analysis.

The underlying etiology of immunosuppression included systemic rheumatic diseases (*n* = 86) and inflammatory diseases (*n* = 21). The most common conditions were systemic vasculitis, idiopathic inflammatory myopathies (IIMs), systemic lupus erythematosus, and rheumatoid arthritis (RA), accounting for 71% (*n* = 76). Connective tissue disease-associated interstitial lung disease (CTD-ILD) was present in 55 patients (51.4%). All patients had a history of long-term corticosteroid use, with a median duration of 4 months and a median prednisolone-equivalent dose of 40 mg/day at PCP onset; 15 patients (14%) were on a dose of less than 20 mg/day. Immunosuppressants had been administered to 68 patients (63.6%), and 9 patients (8.4%) had received either rituximab or tumor necrosis factor-α antagonists. Notably, only 8 patients (7.5%) were on prophylacticTMP-SMX.

Patients’ characteristics at ICU admission are summarized in **Table 1**. The mean APACHE II and SOFA scores were 17.4 and 6.8, respectively. The mean P/F was 167 mmHg, and 82 (76.6%) patients required MV. Pulmonary coinfection was detected in 46 (43.0%) patients. Septic shock was present in 26 patients (24.3%), and was significant associated with coinfection (OR, 4.09; 95% CI, 1.447–17.688; *p* = 0.004). Relevant laboratory parameters, including complete blood count, serum albumin, serum creatinine, 1, 3-ß-D-glucan, lactate dehydrogenase (LDH) are presented in **Table 1**. A CD_4_^+^ T-cell count < 200 cells/L was observed in 94 patients (87.9%). The mean cycle threshold (Ct) value by quantitative PCR was 26.3. The most frequent HRCT finding was bilateral diffuse ground glass opacity (GGO; *n* = 92), followed by consolidation (*n* = 22), septal thickening (*n* = 16), traction bronchiectasis/bronchiolectasis (TBE; n = 9), honeycombing (*n* = 8), pneumomediastinum (*n* = 5) and pneumothorax (*n* = 3). The mean HRCT fibrosis score was 218 ± 40.

**Table 1:** Baseline characteristics of severe *Pneumocystis* pneumonia in patients with autoimmune and inflammatory diseases.

Characteristic	All (*n* = 107)	Survivals (*n* = 46)	Nonsurvivals (*n* = 61)	*P* value
Age, years	52 ± 17	49 ± 19	53 ± 16	0.039
Female	68(63.6)	29(63.0)	39(63.9)	0.318
Source--emergency department	90(84.1)	36(78.3)	54(88.5)	0.819
LOS before admission to ICU, days	2.0(1.1,3.3)	2.0(1.3,3.3)	2.0(1.1,3.5)	0.740
Comorbidities				
Hypertension	27(25.2)	12(26.1)	15(24.6)	0.773
Chronic cardiac insufficiency	9(8.4)	3(6.5)	5(8.2)	0.648
Diabetes	21(19.6)	11(23.9)	10(16.4)	0.583
CKD stage 3/4/5	23(21.5)	9(19.6)	14(23.0)	0.672
Type of AID				
Systemic rheumatic disease	86(80.4)	38(82.6)	48(78.7)	0.332
Systemic lupus erythematosus	17(15.9)	8(17.4)	9(14.8)	0.712
Idiopathic inflammatory myopathies	24(22.4)	7(15.2)	17(27.9)	0.043
Systemic vasculitis	27(25.2)	12(26.1)	15(24.6)	0.860
Rheumatoid arthritis	8(7.5)	5(10.9)	3(4.9)	0.247
Others ^a^	10(9.3)	6(13)	4(6.6)	0.254
Inflammatory diseases ^b^	21(19.6)	12(26.1)	9(14.8)	0.869
CTD-ILD	55(51.4)	17(37.0)	37(60.7)	0.027
Specific therapy for AID at PCP onset				
Glucocorticoid combined with IS	68(63.6)	24(52.2)	44(72.1)	0.036
Duration of steroid, months	4.0(2.0,17.5)	3.8(2.0,17.5)	4.1(1.0,18.0)	0.165
Prednisolone-equivalent dose, mg/d	40(20,45)	38(18,45)	40(20,40)	0.880
Prednisolone-equivalent dose < 20 mg/d	15(14.0)	5(10.9)	10(16.4)	0.415
PCP prophylaxis	8(7.5)	4(8.7)	4(6.6)	0.693
Disease severity at ICU admission				
APACHE II score	17.4 ± 5.6	16.9 ± 6.9	17.7 ± 4.7	0.073
SOFA score	6.8 ± 2.5	6.5 ± 2.7	7.1 ± 2.5	0.041
PaO_2_/FiO_2_ ratios, mmHg	167 ± 56	202 ± 68	134 ± 26	< 0.001
Septic shock	26(24.3)	10(21.7)	16(26.2)	0.713
Mechanical ventilation	82(76.6)	31(67.4)	51(83.6)	0.015
Pulmonary coinfection at PCP onset	46(43.0)	13(28.3)	33(54.1)	0.008
Cytomegalovirus	38(35.5)	11(23.9)	27(44.3)	0.029
Aspergillus	21(19.6)	5(10.9)	16(26.2)	0.048
Bacterium	7(6.5)	4(8.7)	3(4.9)	0.434
Laboratory tests at ICU admission				
WBC count, 10^9^ cells/L	7.3 ± 3.8	7.1 ± 4.3	7.5 ± 4.5	0.212
Neutropenia	6(5.6)	2(4.3)	4(6.6)	0.577
Platelet, 10^9^ cells/L	99(66,151)	102(78, 158)	95(66,138)	0.121
Lymphocyte, 10^6^ cells/L	414(207,567)	460(275, 832)	370(175, 539)	0.027
CD_4_*^+^* T cell, 10^6^ cells/L	138(62,185)	148(89,219)	108(65,178)	0.014
Serum creatinine, µml/L	113 ± 39	109 ± 45	121 ± 35	0.679
Serem albumin, g/L	29 ± 3	30 ± 5	29 ± 4	0.151
1,3-β-D-glucan, pg/mL	465(241,613)	454(282,639)	478(235,629)	0.280
LDH, IU/L	667(401,977)	647(414,1040)	684(423,913)	0.507
Ct value by qPCR	26.3 ± 5	26.5 ± 7	25.7 ± 9	0.337
HRCT fibrosis score on ICU admission	218 ± 40	209 ± 42	223 ± 45	0.316
Barotrauma on ICU admission	8(7.5)	2(4.3)	6(9.8)	0.285

The value is expressed as no. of patients (%) or mean ± SD or median (IQR).

AID, autoimmune and inflammatory diseases; APACHE, acute physiology and chronic health evaluation; CKD, chronic kidney disease; Ct, cycle threshold; CTD-ILD, connective tissue disease-associated interstitial lung disease; HRCT, high-resolution CT; IS, immunosuppressants; ICU, intensive care unit; LDH, lactate dehydrogenase; LOS, length of stay; PCP, *Peumocystis* pneumonia; qPCR, quantitative PCR; SOFA, sequential organ failure assessment; WBC, white blood cell.

***^a^*** including Adult onset Still’s disease (*n* = 4), Sjogren’s disease (*n* = 4), Undifferentiated connective tissue disease (*n* = 2), respectively.

***^b^*** including primary nephrotic syndrome (*n* = 10), pemphigoid (*n* = 4), myasthenia gravis (*n* = 3), autoimmune encephalitis (*n* = 2) and ulcerative colitis (*n* = 2).

### Treatment, early follow-up and outcome

All patients were initially treated with an anti-PCP regimen of standard-dose oral TMP/SMX (12–15 mg/kg/day) combined with glucocorticoids. Twenty-eight patients (26.2%) received an initial combined anti-PCP regimen with caspofungin or clindamycin. The median interval from symptom onset to treatment initiation was 5.5 days. The details of empirical anti-infective management targeting other potential pathogens are as follows: All patients received antibacterial therapy. In addition, 88 patients (82.2%) were administered anti-cytomegalovirus agents (ganciclovir or foscarnet), and 20 (18.7%) received caspofungin or voriconazole for anti-aspergillus therapy. Laboratory parameters obtained during early follow-up are summarized in **Table 2**. Follow-up HRCT scans revealed evidence of worsening fibrosis (score: 263 ± 40), which presented with features of architectural distortions (such as irregular linear opacities, reticulations and TBE) in 66 patients (61.7%), and significant parenchymal consolidation in 48 (44.9%). Extent average score of HRCT finding at ICU admission and during early follow-up are presented in **Supplementary Table 2**. Interobserver agreement for HRCT assessment was good (Spearman rank correlation coefficient, 0.72; *p* < 0.01). Initial treatment failure occurred in 52 patients (48.6%). Among these patients, 27 received salvage regimens with second-line anti-PCP agents, including clindamycin plus TMP/SMX (*n* = 9) and caspofungin plus TMP/SMX (*n* = 18). Anti-infective regimens were adjusted in 15 patients: 4 cases had omitted aspergillosis coverage in the initial empirical therapy, 2 cases had omitted cytomegalovirus coverage, and 9 cases were targeted at newly isolated pathogens (4 *Klebsiella pneumoniae* and 5 *Acinetobacter baumannii*). Additionally, 16 underwent prone position ventilation, and 4 required extracorporeal membrane oxygenation (ECMO). Among those with initial treatment failure, 40 patients died, including all who underwent ECMO.

**Table 2:** Clinical course between survivors and nonsurvivors: treatment, laboratory tests, imaging and complications during the ICU stay.

Characteristic	All (*n* = 107)	Survivals (*n* = 46)	Nonsurvivals (*n* = 61)	*P* value
Anti-PCP treatment regimen				
Symptom onset until treatment, days	5.5(3.0,8.3)	5.5(3.0,8.2)	5.4(3.1,8.5)	0.867
Initial combination therapy	28(26.2)	13(28.3)	15(24.6)	0.807
Initial treatment failure	52(48.6)	12(26.1)	40(65.6)	< 0.001
Inappropriate initial empirical therapy	8(7.5)	2(4.3)	6(9.8)	0.285
Omission of aspergillosis coverage	6(5.6)	2(4.3)	4(6.6)	0.623
Omission of Cytomegalovirus coverage	3(2.8)	1(2.2)	2(3.3)	0.732
HRCT findings in the early follow-up *^a^*				
Time interval from first CT scan, days	8 ± 2	9 ± 2	8 ± 2	0.134
HRCT fibrosis score	263 ± 40	230 ± 48	292 ± 56	< 0.001
Disappearance in GGO	21(19.6)	15(32.6)	6(9.8)	0.003
Architectural distortions	66(61.7)	23(50.0)	43(70.5)	0.016
Significant consolidation	48(44.9)	13(28.3)	35(57.4)	0.007
Laboratory results in the early follow-up				
WBC count, 10^9^ cells/L	8.3 ± 3.5	8.2 ± 2.9	8.3 ± 4.8	0.757
Lymphocyte, 10^6^ cells/L	290(183,558)	310(269,622)	250(157,506)	0.007
CD_4_*^+^* T cell, 10^6^ cells/L	105(67,183)	152(79,188)	81(50,137)	0.001
Platelet, 10^9^ cells/L	94(57,139)	107(63,128)	93(56,148)	0.221
Serum creatinine, µmol/L	123 ± 53	113 ± 47	129 ± 57	0.837
1,3-β-D-glucan, pg/mL	245(158,326)	217(118,307)	235(166,316)	0.220
LDH, IU/L	478(227,761)	430(265,711)	495(253,751)	0.355
Complications during the ICU stay				
ICU-acquired infections	48(44.9)	15(32.6)	33(54.1)	0.028
Newly developed organ failure	51(47.7)	15(32.6)	36(59.0)	0.007
Septic shock	45(37.4)	12(26.1)	33(54.1)	0.002
Failure to NIV or HFNC, n (%)	15(14.0)	3(6.5)	12(19.7)	0.088
AKI requiring CRRT	14(13.3)	5(10.9)	9(14.8)	0.555
Newly barotrauma *^b^*	17(15.9)	3(6.5)	14(23.0)	0.027

The value is expressed as no. of patients (%) or mean ± SD or median (IQR).

AKI, acute kidney injury; CRRT, continuous renal replacement therapy; GGO, ground glass opacity; HFNC, high-flow nasal cannula oxygen; HRCT, high-resolution CT; ICU, intensive care unit; LDH, lactate dehydrogenase; NIV, non-invasive ventilation; PCP, *Pneumocystis* pneumonia; WBC, white blood cell.

*^a^* HRCT follow-up was performed in 103 cases; 4 cases were not followed up as they were transferred out within a week.

*^b^* Including pneumomediastinum (*n* = 4) and pneumothorax (*n* = 13).

During ICU hospitalization, 15 patients initially managed with NIV or HFNC required endotracheal intubation after a median of 4.4 days. Newly organ failure developed in 51 patients (47.7%), including septic shock (*n* = 45) and acute kidney injury necessitating renal replacement therapy (*n* = 14). Other complications included ICU-acquired infections (*n* = 48), gastrointestinal bleeding (defined as a hemoglobin decrease >10g/L within 24 hours; *n* = 11), acute coronary syndrome (*n* = 15), new-onset pneumothorax (*n* = 13) and pneumomediastinum (*n* = 4). Among 28 patients with rapidly progressive pulmonary fibrosis (PPF), 11 received high-dose methylprednisolone (2 mg/kg for 7 to14 days, tapered to 1 mg/kg for 7 days, then reduced to 0.5 mg/kg for 7 days, until resumed the pre-PCP maintenance dose); clinical improvement was observed in 4 patients with suspected organizing pneumonia and 1 with progressive CTD-ILD.

The 28-day ICU mortality was 57.0% (*n* = 61). Overall, 65 (60.7%) patients died during hospitalized. The median hospital LOS was 28.0 (16.5–39.5) days.

### Predictors of 28-day ICU mortality

In univariate analysis, compared to survivors, nonsurvivors were significantly older and had a higher prevalence of IIMs, CTD-ILD, immunosuppressant use, pulmonary coinfection at PCP onset, and greater illness severely (reflected by higher SOFA scores and lower P/F; all *p* < 0.05; **Table 1**). During early follow-up, nonsurvivors exhibited higher rates of initial treatment failure and higher fibrosis scores on HRCT; whereas survivors were more likely to show GGO resolution (all *p* < 0.05; **Table 2**). During the ICU stay, nonsurvivors also experienced more complications, including ICU-acquired infections, new-onset shock, and barotrauma (all *p* < 0.05; **Table 2**). No significant differences were observed in most laboratory parameters at admission or during early follow-up between groups, except for lymphocyte and CD_4_^+^ T-cell counts (**Table 2**).

**Figure 1:**
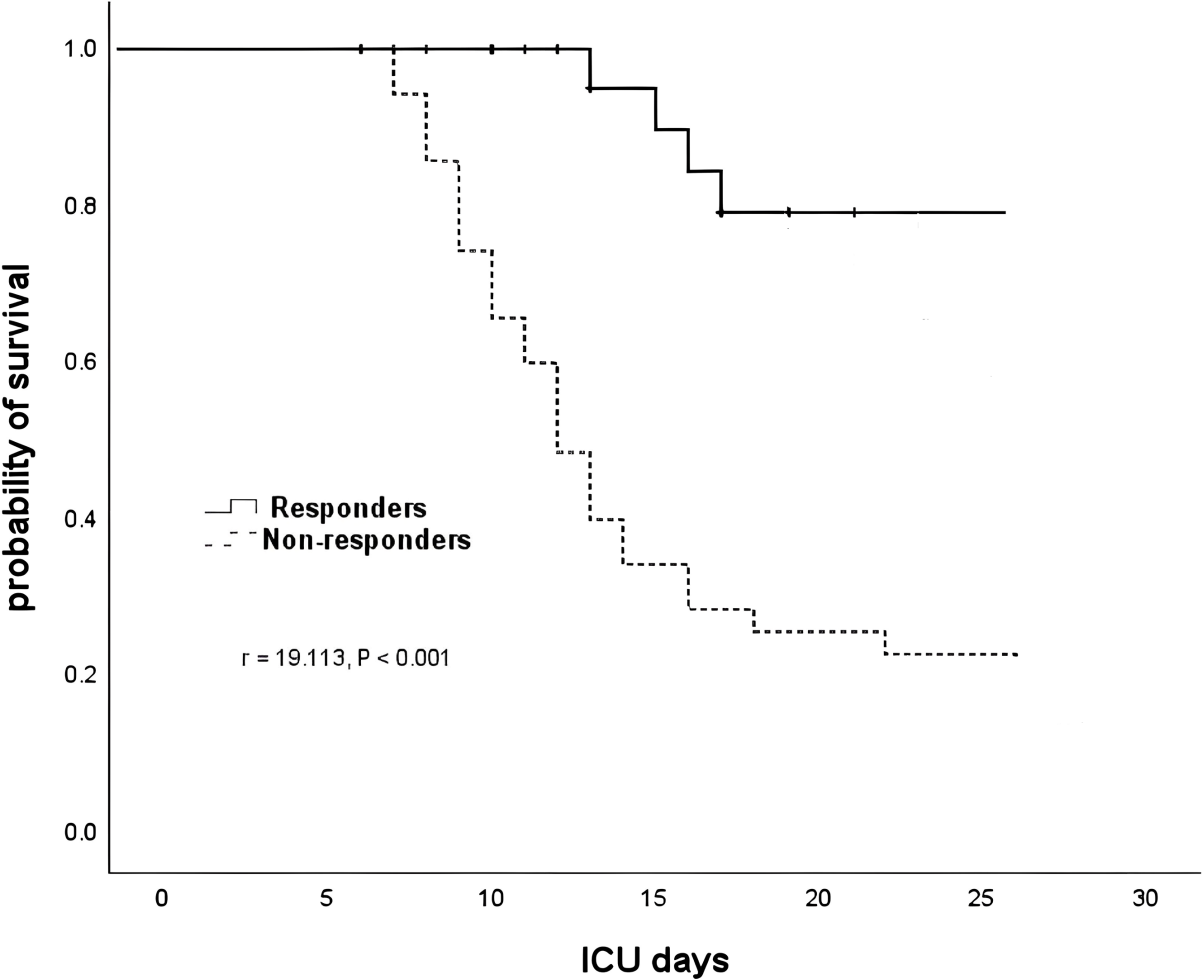
Kaplan-Meier analysis of 28-day ICU survival according initial treatment response.

Multivariable analysis identified severe hypoxemia (P/F < 100 mmHg) at ICU admission, initial treatment failure, high HRCT fibrosis scores (> 260) and persistently low CD_4_^+^ T-cell counts (< 100 cells/μL) during early follow-up, and new-onset shock during the ICU stay as independent predictors of 28-day ICU mortality (**Table 3**). The prediction model obtained good discrimination (C-statistic, 0.903, 95% CI, 0.845–0.964, *p* < 0.001) and calibrated (Hosmer-Lemeshow *X ^2^* statistic of 4.668; *p* = 0.817). Kaplan–Meier analysis showed significantly worse survival in patients with initial treatment failure (r = 19.113, *p* < 0.001; **Figure 1**).

**Table 3:** Multivariate logistic regression analysis of risk factors for 28-day ICU mortality ^*^.

Variables	Odds ratio	95% Confidence interval	*P* value
PaO_2_/FiO_2_ < 100 mmHg on ICU admission	8.119	1.543-32.871	0.007
Initial treatment failure	10.591	1.926-45.768	0.013
HRCT fibrosis score >260 during early follow-up	5.354	1.513-19.756	0.008
New-onset shock during the ICU stay	2.147	1.237-39.157	0.018
CD_4_^+^ T-cell <100 cells/μL during early follow-up	4.137	1.219-62.152	0.021

^*^ Variables in the multivariate logistic regression analysis included age, type of autoimmune and inflammatory diseases, underlying connective tissue disease-associated interstitial lung disease, glucocorticoid combined with immunosuppressants, SOFA scores, PaO_2_/FiO_2_ ratios at admission, pulmonary coinfection, HRCT score at early follow-up, initial treatment failure, and CD4+ T cell count at the early follow-up, ICU-acquired infections, failure to non-invasive ventilation or non-invasive ventilation, new-onset barotrauma and new-onset shock during the ICU stay.

### Subgroup analysis of patients with initial treatment failure

In the univariate analysis, several baseline factors were associated with initial treatment failure: underlying CTD-ILD, IIMs, pulmonary co-infections, a high HRCT fibrosis scores at PCP onset, a low P/F ratio, and a low Ct value by qPCR (all, *p* < 0.05; **Supplementary Table 3**). Multivariate analysis identified underlying CTD-ILD (OR, 3.241; 95% CI, 1.337-25.147; *p* = 0.004), P/F < 100 mmHg (OR, 6.873; 95% CI, 1.746–31.048; *p* = 0.006), and pulmonary co-infection (OR, 5.419; 95% CI, 1.565-38.759; *p* = 0.008) as independently risk factors for initial treatment failure.

## Discussion

To our knowledge, this is the first prospective study focusing on severe PCP in AID patients. We demonstrated that severe hypoxemia at PCP onset and early follow-up parameters—including initial treatment failure, high HRCT fibrosis scores, and persistent low CD_4_^+^ T-cell counts—were associated with poor outcomes. These findings provide clinicians with valuable insights for monitoring and potential intervention.

Among AID patients with severe PCP, we observed a 28-day ICU mortality rate of 57%, which is higher than the 32-49% reported in general AID-PCP populations (Aoki et al., 2009; Chen et al., 2015; Kageyama et al., 2019; Shi et al., 2025), yet consistent with mortality exceeding 50% in non-HIV PCP cases with respiratory failure (Roux et al., 2014; Schmidt et al., 2018; Gaborit et al., 2019; McMullan et al., 2024). Although various prognostic factors have been proposed for non-HIV PCP, including advanced age, coinfection, hypoxemia, underlying CTD-ILD, pneumothorax, lymphocytopenia, hypoalbuminemia and elevated LDH (Aoki et al., 2009; Chen et al., 2015; Schmidt et al., 2018; Gaborit et al., 2019; Kageyama et al., 2019; Wang et al., 2021; Shi et al., 2025), the literatures remain inconsistent due to heterogeneity in population, era, diagnostic criteria, disease severity, and inherent limitations of retrospective designs (Wang et al., 2021; Ibrahim et al., 2023). To address this, we prospectively enrolled a homogeneous cohort of AID patients with similar disease stage and severity, thereby mitigating these sources of bias.

Unlike previous reports emphasizing baseline factors, our study is the first to underscore the prognostic value of early follow-up parameters. We observed an initial treatment failure rate of 48%, consistent with previous reports (40–44%) (Ko et al., 2014; Ko et al., 2019), which served as an independent predictor of outcome. In clinical practice, differentiating the causes of initial treatment failure—whether due to PCP progression, drug toxicity, pulmonary injury following anti-PCP therapy, or coinfection—remains challenging (Ko et al., 2014; Ko et al., 2019). Our subgroup analysis found that severe hypoxemia at ICU admission, pre-existing CTD-ILD, and pulmonary coinfection were associated with treatment failure. In contrast, neither fungal burden (as indicated by Ct values) nor combination anti-PCP therapy showed a significant associated. This suggests that, within the framework of standard anti-PCP, initial treatment failure may not be attributable to PCP itself.

Contrary to previous literature reports (Kageyama et al., 2019; Wang et al., 2021; Shi et al., 2025), we only identified that underlying CTD-ILD and coinfection (particularly CMV pneumonia)were associated with high mortality in univariate analysis, but not in multivariate analysis, possibly due to the limited sample size or potential confounding factors. However, both conditions were independent predictors of initial treatment failure, which in turn was strongly correlated with elevated mortality. Thus, heightened vigilance and timely assessment of treatment response are warranted for the high-risk subgroup of patients with pre-existing CTD-ILD and/or pulmonary coinfection. Unfortunately, owing to the relatively small sample size, we didn’t identify a significant association between specific pathogens involved in coinfection and initial treatment failure (**Supplementary Table 3**), highlighting the need for further large-scale cohort investigations.

The incidence of coinfection in our cohort (43%) exceeded that reported in previous non-HIV PCP studies (25-34%) (Ko et al., 2014; Roux et al., 2014; Chen et al., 2015), which may reflect an increased susceptibility to various pathogens due to immune dysfunction inherent to AID and their immunosuppressive therapy. Comprehensive microbiological workup is essential for the early diagnosis and management of coinfection, particularly in critically ill patients with septic shock, who are frequently complicated by coinfection. Undeniably, most enrolled patients were treated with combined anti-infective regimens, leading to a low rate of inappropriate initial antimicrobial therapy in this cohort. Nevertheless, such broad-spectrum combination strategies also carry the risk of excessive drug exposure. Future studies are therefore warranted to optimize empirical antimicrobial regimens for this patient population. In particular, it remains necessary to further clarify whether septic shock is an independent risk factor for coinfection, which would facilitate targeted pathogen screening and the formulation of individualized empirical anti-infective strategies. Notably, our observations indicated that fibroproliferation extent during follow-up HRCT, rather than initial imaging characteristics, was correlated with prognosis. The predictive value of HRCT manifestations for clinical outcomes remains incompletely characterized. Although some studies have linked initial radiological patterns, such as crazy-paving GGO, mixed consolidations, and pneumothorax, to poor outcomes (Mu et al., 2016; Kumagai et al., 2019; Liu et al., 2019), others have not established a clear relevance of imaging features (Tokuda et al., 2008; Tasaka et al., 2010). These discrepancies may stem from confounding factors affecting baseline imaging, such as heterogeneity in disease stage, concurrent coinfection and underlying CTD-ILD. In this prospective study, serial HRCT evaluation revealed interstitial abnormalities—including architectural distortion, increased consolidation, fibrosis or aerocysts changes—in more than half of patients, and non-survivors exhibited significantly higher fibrosis scores. Previous reports have described irregular linear opacities, reticulations, and TBE in over one-third of non-HIV PCP patients, with these architectural distortions being implicated in PCP-related mortality (Kuroda et al., 2016; Maschmeyer et al., 2016). Several observational studies have also noted that patients with CTD-ILD are more susceptible to PPF following PCP (Kuroda et al., 2016; Shi et al., 2025). The high proportion of CTD-ILD patients in our cohort may explain the pronounced fibrotic progression observed. The pathogenesis of PCP-associated fibrosis remains poorly understood. Either PCP per se or inflammatory-immune dysregulation secondary to anti-PCP treatment may serve as causes or promoters of fibrosis (Saldana et al., 1989; Iturra et al., 2018; Gharamti et al., 2021; Yu et al., 2024). Previous studies have reported that later expansion of lethal interstitial pneumonia may be a characteristic feature of PCP in AID patients with pre-existing CTD-ILD. This can occur even after the infection has microbiologically resolved, as indicated by low serum 1, 3-β-D-glucan levels or negative PCR results (Kuroda et al., 2016; Shi et al., 2025). Our results align with these observations and imply that the mechanisms driving fibrosis are likely more complex in AID patients with PCP, especially those with concomitant CTD-ILD, warranting further investigation.

The effect of CAT on survival in non-HIV PCP remains controversial (Wang et al., 2021, , Lemiale et al., 2013; Ishikawa et al., 2021). Existing studies are constrained by limitations such as retrospective designs, small sample sizes, variations in dosage and timing of corticosteroids, and heterogeneous cohorts. A meta-analysis suggested that CAT may improve outcomes in non-HIV PCP with respiratory failure (Ding et al., 2020). The inability of our study to evaluate the effect of CAT on prognosis—as all patients were indicated for CAT based on severe hypoxemia, underlying corticosteroid-maintained diseases, and high fungal burden. However, the only recent prospective study in non-HIV population reported that patients randomized to CAT had significantly lower intubation rates following respiratory deterioration (10.1% vs. 26.1%, *p* = 0.020) and lower 90-day mortality (28.0% vs. 43.2%, *p* = 0.022), suggesting a potential benefit of CAT in improving respiratory function and long-term prognosis (Lemiale et al., 2025). Published case reports documented instances in RA patients with severe PCP where respiratory function deteriorated under treatment with TMP-SMX alone yet showed improvement following the administration of high-dose methylprednisolone (Gochi et al., 2014). This lends support to the hypothesis that CAT may provide a protective effect in AID patients with severe PCP, particularly among those in whom the infection risks precipitating an acute exacerbation of pre-existing ILD. It is noteworthy that acute respiratory distress syndrome develops in approximately half of PCP cases, often resulting in persistent pulmonary dysfunction (Maschmeyer et al., 2016). Consequently, further investigation is warranted to elucidate the mechanisms underlying the respiratory improvement associated with corticosteroids and to determine the specific subgroups within the highly heterogeneous non-HIV PCP population, or the precise clinical stages, for which CAT may be most beneficial.

In line with existing literature (Gaborit et al., 2019; Kageyama et al., 2019; Wang et al., 2021), non-survivors in our cohort exhibited persistent lymphocytopenia or low CD_4_^+^ T-cell counts during early follow-up. This sustained immunosuppression likely contributed to the increased incidence of nosocomial infections and septic shock, with the latter also identified as a risk factor for mortality. Currently, there is no consensus on PCP prophylaxis for AID patients. Although guidelines recommend prophylactic TMP/SMX for non-HIV patients on long-term corticosteroid therapy at prednisone-equivalent doses exceeding 20 mg/day (), fewer than 10% of our patients received such prophylaxis, despite the majorit**y** being treated with high-dose corticosteroid and having CD_4_^+^ T-cell counts below 200 cells/μL. These findings underscore an urgent need to strengthen the implementation of PCP prophylaxis in high-risk AID populations to reduce morbidity and mortality.

Our study had several limitations. First, as a single-center investigation, our findings necessitate validation through future multicenter prospective studies. Second, we did not monitor TMP/SMX drug concentrations or perform serial assessments of fungal burden, precluding an evaluation of their impact on the initial treatment response. Nevertheless, existing evidence indicates that disease severity, rather than TMP/SMX dosage (Ohmura et al., 2019; Tritle et al., 2021) or PCR negative conversion (Roger et al., 1998; White et al., 2018), serves as the primary determinant of treatment failure. Third, the relatively high proportion of patients with CTD-ILD or coinfection in this cohort may have introduced bias to pulmonary imaging evaluation; In fact, these characteristics represent key clinical distinctions between patients with AID-associated PCP and other non-HIV immunocompromised individuals. Fourth, although QNAT for CMV in BALF reflects pulmonary viral replication, a consensus on the viral load threshold for diagnosis CMV pneumonia has not yet been reached. In present study, patients with a presumptive diagnosis of CMV pneumonia exhibited relatively high viral loads (median: 2.75 × 10^5^ copies/ml, results not shown), suggesting a higher likelihood of infection. Fifth, to ensure the integrity of evaluable data, patients with an ICU-LOS of less than 7 days were excluded. These individuals may have experienced rapid clinical improvement or deterioration, potentially introducing bias to the prognostic analysis. Finally, the limited sample size restricted to fully adjust for potential confounders (e.g., type of AID, underlying ILD, combination anti-PCP regimens and specific pathogens of coinfection); therefore, our results should be interpreted with caution.

## Conclusion

In this prospective cohort of AID patients with severe PCP, severe hypoxemia at admission and early follow-up parameters, especially initial treatment failure and high HRCT fibrosis scores, were correlated with poor outcome, and warrant further validation through multicenter prospective studies. Future investigations into the mechanisms of treatment failure and pulmonary fibrosis may help identify subpopulations most likely to benefit from CAT and improve outcomes in non-HIV PCP with respiratory failure.

## Data Availability

The original contributions presented in the study are included in the article/**Supplementary Material**. Further inquiries can be directed to the corresponding author.
