# Supplementary material for: Clinical course, treatment and outcome of severe pneumocystis pneumonia in patients with autoimmune or inflammatory diseases: a prospective study
Source: Front Cell Infect Microbiol. 2026 May 27;16:1715992. doi: 10.3389/fcimb.2026.1715992 (PMC13250780; doi:10.3389/fcimb.2026.1715992)
Supplement: Supplementary file 1 [file Table1.docx]

**Supplementary Table S1**. Microbiological tests in the standard procedures

| Pathogen | Technique | Sample | Microbiological Tests |
| --- | --- | --- | --- |
| Bacteria | Bacterial culture | BALF/TA | Bacterial culture on appropriate media with bacterial identification by MALDI-TOF mass spectrometry |
|  | Smear | BALF/TA | Acid-fast staining for *Mycobacterium tuberculosis* |
|  | Antigen detection | Urine | *Legionella* and pneumococcal antigen |
|  | Multiplex PCR | BALF | *Legionella pneumophilia*, *Mycoplasma pneumoniae*, *Chlamydia pneumoniae*, *Mycobacterium tuberculosis* |
| Viruses | Multiplex PCR | BALF | Influenza A/B, human rhinovirus, adenovirus, respiratory syncytial virus (RSV), human metapneumovirus (HMPV), cytomegalovirus (CMV), Epstein-Barr virus (EBV) |
|  | Antigen detection | NP Swab | Influenza A/B antigen |
| Fungi | Smear | BALF/TA | India ink staining for *Cryptococcus* |
|  |  |  | Methemamine silver staining for *Pneumocystis jirovecii* |
|  | Multiplex PCR | BALF | *Pneumocystis jirovecii* |
|  | Fungal culture | BALF/TA | Fungal culture on appropriate media with fungal identification by MALDI-TOF mass spectrometry |
|  | Antigen detection | Blood | Galactomannan and 1,3- β-D glucan antigen |
|  |  | BALF | Galactomannan and cryptococcal antigen |

Abbreviations: BALF, bronchoalveolar lavage fluid; MALDI-TOF, matrix-assisted laser desorption/ ionization-time of flight; NP, nasopharyngeal; PCR, polymerase-chain-reaction; TA, tracheal aspirate

**Supplementary Table S2**. Extent average score of HRCT finding on ICU admission and during the early follow-up^*^

|  | 28d-Survivors  (*n* = 46) | Nonsurvivors  (*n* = 61) | *P* value |
| --- | --- | --- | --- |
| HRCT findings at ICU admission |  |  |  |
| Total fibrosis score | 209±42 | 223±45 | 0.316 |
| (1) Spared area | 9±3 | 8±5 | 0.337 |
| (2) GGO without TBE | 73±15 | 70±14 | 0.173 |
| (3) Consolidation without TBE | 8±4 | 12±5 | 0.253 |
| (4) GGO with TBE | 8±7 | 9±6 | 0.588 |
| (5) Consolidation with TBE | 3±1 | 2±1 | 0.255 |
| (6) Honeycombing ^a^ | 0.3±1.2 | 0.9±2.2 | 0.211 |
| HRCT findings during the early follow-up |  |  |  |
| Total fibrosis score | 230±48 | 292±56 | < 0.001 |
| (1) Spared area | 33±11 | 10±3 | 0.001 |
| (2) GGO without TBE | 37±13 | 22±12 | 0.104 |
| (3) Consolidation without TBE | 11±7 | 25±6 | 0.039 |
| (4) GGO with TBE | 10±8 | 20±7 | 0.078 |
| (5) Consolidation with TBE | 8±5 | 22±3 | 0.030 |
| (6) Honeycombing ^b^ | 0.5±1.2 | 1.2±2 | 0.241 |

^*^Average scores for overall are expressed percentage of lung parenchyma

Abbreviations: GGO, ground glass opacity; ICU, intensive care unit; HRCT, high-resolution CT; TBE, traction bronchiectasis or bronchiolectasis

^a^ There were only 8 cases with positive findings.

^b^ There were only 18 cases with positive findings.

**Supplementary Table S3**. Baseline characteristics of 107 patients according to the initial treatment response

|  | Responders  (*n* = 55 ) | Non- Responders  (*n* =52) | *P* value |
| --- | --- | --- | --- |
| Age, years | 51±15 | 52±17 | 0.879 |
| Female | 31(56.4) | 37(71.2) | 0.347 |
| Source: emergency department | 40(72.7) | 50(96.2) | 0.186 |
| LOS before admission to ICU, days | 2.0 (1.2,5.5) | 2.0 (1.0,4.0) | 0.676 |
| Comorbidities |  |  |  |
| Hypertension | 12(21.8) | 15(28.8) | 0.403 |
| Chronic cardiac insufficiency | 5(9.1) | 4(7.7) | 0.794 |
| Diabetes | 8(14.5) | 13(25) | 0.174 |
| Chronic kidney disease stage 3/4/5 | 9(16.4) | 14(26.9) | 0.184 |
| Type of AID |  |  |  |
| Systemic rheumatic disease | 40(72.7) | 46(88.5) | 0.041 |
| Systemic lupus erythematosus | 8(14.5) | 9(17.3) | 0.696 |
| Idiopathic inflammatory myopathies | 8(14.5) | 16(30.8) | 0.044 |
| Systemic Vasculitis | 11(20.0) | 16(30.8) | 0.200 |
| Rheumatoid arthritis | 6(10.9) | 2(3.8) | 0.165 |
| Others | 7(12.7) | 3(5.8) | 0.217 |
| Inflammatory diseases | 15(27.3) | 6(11.5) | 0.041 |
| CTD-ILD | 20(36.4) | 35(67.3) | 0.001 |
| Specific therapy for AIDs at PCP onset |  |  |  |
| Glucocorticoid combined with IS | 32(58.2) | 36(69.2) | 0.235 |
| Duration of steroid, months | 3.8(2.3,33.0) | 4.0(2.0,24.0) | 0.758 |
| Prednisolone-equivalent dose, mg/d | 40(15,50) | 40(20,45) | 0.618 |
| PCP prophylaxis | 5(9.1) | 3(5.8) | 0.514 |
| Severity of illness at ICU admission |  |  |  |
| APACHE II score | 16.9±6.3 | 17.5±5.3 | 0.185 |
| SOFA score | 6.7±2.9 | 7.1±2.1 | 0.101 |
| PaO_2_/FiO_2_ ratios, mmHg | 186 ± 36 | 105 ±30 | <0.001 |
| Septic shock | 10(18.2) | 16(30.8) | 0.129 |
| Mechanical ventilation | 40(72.7) | 42(80.8) | 0.326 |
| Pulmonary coinfection at PCP diagnosis | 15(27.3) | 31(59.6) | 0.001 |
| Cytomegalovirus | 15(27.3) | 23(44.2) | 0.067 |
| Aspergillus | 7(12.7) | 14(26.9) | 0.065 |
| Bacterium | 3(5.5) | 4(7.7) | 0.640 |
| Inappropriate initial empirical therapy | 3(5.5) | 5(9.6) | 0.413 |
| Omission of cytomegalovirus coverage | 1(1.8) | 2(3.8) | 0.525 |
| Omission of aspergillosis coverage | 2(3.6) | 4(7.7) | 0.362 |
| Laboratory tests at ICU admission |  |  |  |
| WBC count, 10^9^ cells/L | 7.1(5.5,8.9) | 8.8(5.1,13.1) | 0.164 |
| Neutropenia | 3 (9.1) | 3(8.6) | 0.940 |
| Lymphocyte, 10^6^ cells/L | 430(231,654) | 414(151,543) | 0.342 |
| Platelet, 10^9^ cells/L | 109(75,154) | 102(69,152) | 0.552 |
| CD4*^+^* T cell, 10^6^ cells/L | 150(73, 219) | 123(64,119) | 0.137 |
| Serum creatinine, µmol/L | 107±37 | 128±42 | 0.258 |
| Serum albumin, g/L | 29 ±5 | 30 ±3 | 0.327 |
| 1,3-β-D-glucan, pg/mL | 454(255,679) | 475(236,630) | 0.397 |
| Lactate dehydrogenase, IU/L | 634(436,951) | 671(408,1099) | 0.067 |
| Ct value by qPCR | 28.4± 6 | 25.3±3 | 0.038 |
| Initial combination anti-PCP therapy | 16(29.1) | 12(23.1) | 0.472 |
| Symptom onset until treatment, days | 5.3(3.0, 8.0) | 5.5(3.0,7.0) | 0.351 |
| HRCT score on ICU admission | 180±48 | 225±37 | 0.013 |
| Barotrauma on ICU admission | 3(5.5) | 5(9.6) | 0.692 |

The value is expressed as no. of patients (%) or mean±SD or median (IQR)

Abbreviations: AID, autoimmune and inflammatory diseases; APACHE, acute physiology and chronic health evaluation; Ct, cycle threshold; CTD-ILD, connective tissue disease-associated interstitial lung disease; HRCT, high-resolution CT; IS, immunosuppessants; ICU, intensive care unit; LOS, length of stay; PCP, *pneumocystis* pneumonia; qPCR, quantitative PCR; SOFA, sequential organ failure assessment; WBC, white blood cell
